# Impact of a 12-Week Multicomponent Training Program with Multiprofessional Support on Body Composition, Metabolic Markers, and Arterial Stiffness in Brazilian Older Women Stratified by Nutritional Status: A Secondary Analysis

**DOI:** 10.3390/nu18081227

**Published:** 2026-04-14

**Authors:** Jordan Hernandez-Martínez, Pablo Valdés-Badilla, Izham Cid-Calfucura, Edgar Vásquez-Carrasco, Lucimere Bohn, Jorge Mota, Cristian Sandoval-Vásquez, Marilene Ghiraldi de Souza Marques, Braulio Henrique Magnani Branco

**Affiliations:** 1 Department of Physical Activity Sciences, Universidad de Los Lagos, Osorno 5290000, Chile; jordan.hernandez@ulagos.cl; 2 Department of Education, Faculty of Humanities, Universidad de La Serena, La Serena 1700000, Chile; 3 Department of Physical Activity Sciences, Faculty of Education Sciences, Universidad Católica del Maule, Talca 3530000, Chile; 4 Sports Coach Career, Faculty of Life Sciences, Universidad Viña del Mar, Viña del Mar 2520000, Chile; 5 Department of Physical Activity, Sports and Health Sciences, Faculty of Medical Sciences, Universidad de Santiago de Chile (USACH), Santiago 8370003, Chile; izham.cid@gmail.com; 6 School of Occupational Therapy, Faculty of Psychology, Universidad de Talca, Talca 3465548, Chile; edgar.vasquez@utalca.cl; 7 Centro de Investigación en Ciencias Cognitivas, Faculty of Psychology, Universidad de Talca, Talca 3465548, Chile; 8 VITALIS Longevity Center, Universidad de Talca, Talca 3465548, Chile; 9 Program in Health Promotion Postdoctoral, Cesumar University (UniCesumar), Maringá 87050-900, Brazil; 10 Faculty of Sport, University of Porto, 4200-450 Porto, Portugal; lucimerebohn@gmail.com; 11 Research Centre of Physical Activity, Health, and Leisure, Laboratory for Integrative and Translational Research in Population Health (ITR), Faculty of Sports, University of Porto, 4200-450 Porto, Portugal; jmota@fade.up.pt; 12 Carrera de Terapia Ocupacional, Facultad de Ciencias de La Salud, Universidad Autónoma de Chile, Temuco 4810101, Chile; cristian.sandoval@ufrontera.cl; 13 Departamento de Medicina Interna, Facultad de Medicina, Universidad de La Frontera, Temuco 4811230, Chile; 14 Program in Health Promotion, Cesumar University (UniCesumar), Maringá 87050-900, Brazil; marileneghiraldi@gmail.com (M.G.d.S.M.); braulio.branco@unicesumar.edu.br (B.H.M.B.)

**Keywords:** arterial stiffness, cardiovascular disease, body fat distribution, healthy aging, multicomponent exercise, visceral adiposity

## Abstract

**Background/Objectives:** this study evaluated the effects of a 12-week multicomponent training (MCT) program combined with multiprofessional interventions (nutritional and psychoeducational) on body composition, lipid profiles, fasting glucose levels, and arterial stiffness in Brazilian older women stratified by nutritional status. **Methods:** thirty-six older women, who were classified as normal weight (n = 8; mean age: 69.2 ± 7.2 years), overweight (n = 13; mean age: 72.1 ± 5.3 years), or obese (n = 15; mean age: 70.3 ± 4.6 years), were included in the study. The outcomes included body fat percentage (BFP), visceral fat level, fat-free mass (FFM), fasting glucose, total cholesterol, high-density lipoprotein cholesterol, low-density lipoprotein cholesterol, triglycerides, and arterial stiffness, as determined by pulse wave velocity (PWV). **Results:** BFP was reduced in all groups (*p* < 0.001), but in the overweight and obese groups, these decreases were greater. Visceral fat level decreased significantly in all groups (*p* < 0.001), with greater decreases in the obese group compared with the normal weight and overweight groups (*p* < 0.001), and greater reductions in the normal weight group compared to the overweight group (*p* < 0.05). A significant reduction in PWV was observed only in the overweight group (*p* < 0.05), while greater improvements were observed in the overweight group compared to the normal weight group (*p* < 0.05) in FFM, lipid profiles, and fasting glucose. **Conclusions:** an MCT combined with multiprofessional intervention effectively reduced BFP and arterial stiffness in Brazilian older women with excess adiposity.

## 1. Introduction

As people grow older, their body composition changes, leading to reductions in fat-free mass (FFM) and bone mineral density and increasing fat mass [1]. These changes tend to accelerate after 50 years of age [1] and are generally more pronounced in women than in men [2]. Moreover, changes in body composition due to aging are associated with adverse effects on lipid metabolism, including increased levels of triglycerides, low-density lipoprotein cholesterol (LDL-c), and total cholesterol [3]. Additionally, advancing age is commonly associated with impairments in glucose homeostasis, as evidenced by elevated blood glucose levels [4]. Collectively, these metabolic and compositional alterations contribute to an elevated cardiovascular risk profile in older people [5,6]. One of the earliest detectable manifestations of this increased risk is arterial stiffness [7], which progressively intensifies with aging because of elastin degradation and the concomitant accumulation of collagen within the arterial wall [8].

Physical inactivity and prolonged sedentary behavior contribute to adverse health outcomes later in life, resulting in an increased prevalence of obesity, elevated visceral fat levels, low muscle mass and low mineral density [9]. These behaviors are associated with metabolic disturbances, including dyslipidemia, hypertension, impaired glucose regulation [10,11], and heightened arterial stiffness [12]. Regular physical activity offers protective benefits against both general and abdominal obesity and is associated with enhanced lipid profiles, reduced blood pressure, reduced circulating glucose levels [13], and decreased arterial stiffness [12]. Current guidelines advocate that older people participate in structured exercise programs encompassing multiple domains of physical fitness at moderate-to-vigorous intensities [14]. Combined exercise strategies, such as combining resistance and aerobic exercise and multicomponent training (MCT), including resistance, aerobic, balance, and flexibility exercises, have demonstrated positive effects on various health-related outcomes in older people with obesity [15,16,17].

A quasi-experimental study conducted in Spain by Subías-Perié et al. [18] involving older people with obesity reported significant increases in high-density lipoprotein cholesterol (HDL-c, *p* < 0.05), together with reductions in glucose levels following a six-month MCT program. Comparable findings were described by Travieso et al. [19] in a randomized controlled trial conducted in Brazilian older people with obesity, where a 12-week MCT intervention resulted in significant reductions in body fat percentage (BFP) compared with an inactive control group. In addition, Hernandez-Martinez et al. [20] evaluated Brazilian older women with hypertension and normotensive counterparts and demonstrated that a 36-week MCT program combined with a multiprofessional intervention resulted in significant improvements in systolic blood pressure in both groups (*p* < 0.05). In hypertensive participants, significant reductions in LDL-c, total cholesterol, and triglyceride concentrations were observed at 12, 24, and 36 weeks (*p* < 0.05). Similarly, a study by Pleticosic-Ramírez et al. [21] conducted in Chilean adults with obesity revealed significant decreases in both total and visceral fat levels after six months of treatment with MCT compared with an inactive control condition. Conversely, a meta-analysis by Ashor et al. [22], including young, adult, and older populations with obesity, indicated that compared with resistance or combined training modalities, aerobic exercise elicited significantly greater reductions in arterial stiffness, as assessed by pulse wave velocity (PWV).

Although previous research has demonstrated the beneficial effects of MCTs on body composition parameters, including BFP, FFM, and visceral fat level, as well as on lipid profiles (HDL-c, LDL-c, total cholesterol, and triglycerides) and glucose metabolism [18,19,20,21], the impact of MCTs combined with multiprofessional interventions on arterial stiffness remains insufficiently explored in older women with obesity [22]. This knowledge gap is particularly relevant in South America, the region with the highest global prevalence of obesity [23], where women are disproportionately affected compared with men [24]. Accordingly, the present study sought to examine the effects of an MCT program integrated with a multiprofessional intervention on body composition, lipid profiles, fasting glucose levels, and arterial stiffness in Brazilian older women stratified by nutritional status. It was hypothesized that the combined intervention would be associated with reductions in total and visceral fat levels, as well as in PWV, with differential responses according to nutritional status.

## 2. Materials and Methods

### 2.1. Study Design

This pilot study utilized a descriptive, comparative design to investigate the immediate effects of a 12-week intervention administered three times per week. Each weekly program consisted of MCT (60 min), a dietary intervention (30–40 min), and psychoeducational activities (30–40 min). The study technique was formulated in accordance with CONSORT principles and consistent with previously established protocols [25]. The experiment was pre-registered prior to participant involvement, and all designated outcomes and statistical analysis were executed in accordance with the a priori protocol. The trial was registered in the United States ClinicalTrials.gov database (NCT07152158, registered on 22 August 2025). Participant recruitment was conducted through multiple channels, including television, radio, and social media. The outcome measures included body composition metrics such as BFP, FFM, and visceral fat level; lipid profiles; fasting glucose levels (glucose, triglycerides, HDL-c, LDL-c, and total cholesterol); and arterial stiffness evaluated through PWV.

All evaluations were conducted in the morning from 9:00 to 11:00 a.m. in the Interdisciplinary Laboratory for Health Promotion Interventions (LIIPS) at Cesumar University in Maringá, Paraná, Brazil. MCT sessions occurred in the morning from 7:00 to 10:00 a.m. (the participants were divided into groups). No musculoskeletal or cardiorespiratory adverse events were documented during the intervention period, and participants reported no pain before outcome assessments or during training sessions.

### 2.2. Participants

A priori sample size estimation was performed using power calculations with G*Power software (version 3.1.9.6; Franz Faul, University of Kiel, Kiel, Germany). Using a two-sided alpha level of 0.05, a statistical power of 90%, and an expected attrition rate of 10%, estimates were derived from data obtained in a pilot study [26]. A mean difference of 0.46 repetitions in the 30 s chair stand test was identified as the minimal clinically relevant change, accompanied by a standard deviation of 3.38 repetitions. The results indicated that 18 total participants are needed to achieve sufficient statistical power.

**Figure 1:**
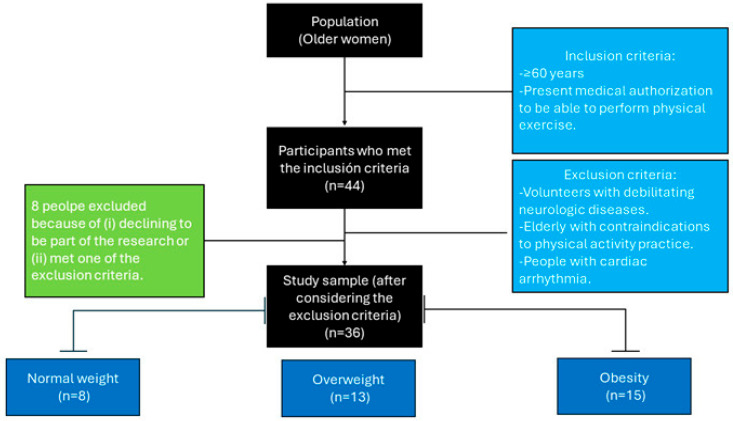
Flowchart of the recruitment process.

The inclusion criteria were being aged 60 years or older and possessing medical authorization to engage in physical exercise. The exclusion criteria included the presence of severe neurological conditions such as Alzheimer’s disease or Parkinson’s disease, absolute contraindications to physical activity, and clinically diagnosed cardiac arrhythmias. A total of 44 individuals were initially invited to participate in the study, but the final sample was composed of 36 women, once 8 individuals opted out of participation or did not satisfy the eligibility requirements. The final 36 participants were classified by nutritional status according to their BFP as indicated by Potter et al. [27] as normal weight (n = 8; <36%), overweight (n = 13; ≥36%), or obese (n = 15; >42%) (Figure 1).

All participants provided consent for data usage and processing by signing an informed consent form, which authorized the use of personal data for scientific purposes. The study was approved by the local Research Ethics Committee of Cesumar University (approval number: 3.373.307) in accordance with the guidelines set forth in Resolution 466/12 of the Brazilian Ministry of Health and the Declaration of Helsinki.

### 2.3. Anthropometry and Body Composition

Participant height (m) was assessed in the standing position using a wall-mounted stadiometer (Welmy R-110^®^, Santa Bárbara d’Oeste, São Paulo, Brazil) with a scale measuring up to 2.2 m and a precision of 0.1 cm. Body composition was assessed using a tetrapolar bioelectrical impedance analysis device (InBody 570^®^, Biospace Co., Ltd., Seoul, Republic of Korea), with a maximum load capacity of 250 kg and a measurement resolution of 100 g. Assessments were conducted following the manufacturer’s standardized procedures, and methodological recommendations were established to improve measurement accuracy and reliability [28].

The parameters obtained included body mass (kg), BFP, visceral fat level, BMI (kg/m^2^), and FFM (kg). Nutritional status classification according to BFP thresholds is defined as follows: <36% indicates normal weight, ≥36% denotes overweight, and >42% signifies obesity [27]. The descriptive characteristics of the study sample are presented in Table 1.

### 2.4. Lipid Profile and Fasting Glucose

Blood sample collection was performed following the guidelines of the Clinical and Laboratory Standards Institute [29]. Participants were provided with standardized precollection instructions, and all venipuncture procedures were conducted at the institution’s Clinical Analysis Laboratory. After sample collection, manual compression was implemented at the puncture site to reduce the likelihood of hematoma formation.

Blood samples were collected in Vacuplast^®^, Elkhart, IN, USA, tubes, which included those containing K2 ethylenediaminetetraacetic acid (EDTA) as an anticoagulant and fluoride/EDTA tubes. Samples designated for serum and plasma separation were centrifuged with a CENTRILAB^®^, Boston, MA, USA, analog centrifuge at 3500 rpm for 15 min at room temperature.

The results of the biochemical analysis revealed markers of the lipid profile and fasting glucose levels, including triglyceride levels, HDL-c levels, LDL-c levels, and total cholesterol levels. All assays were conducted utilizing Gold Analisa^®^, diagnostic kits (Belo Horizonte, Minas Gerais, Brazil) on a URIT 8021^®^ Belo Horizonte, Brazil, semiautomated biochemical and turbidimetric analyzer (MHLab). Each parameter was analyzed in triplicate to ensure analytical reliability.

### 2.5. Arterial Stiffness

Arterial stiffness was assessed in accordance with the methodological recommendations described elsewhere by Bohn et al. [30]: PWV, using photoplethysmography, which records arterial pulse waveforms at the level of the pulpal arteries located in the distal phalanges of the fingers and toes. During the assessment, participants were positioned supine and relaxed, and the resting heart rate was recorded at the beginning of the procedure, per the manufacturer’s guidelines.

A minimum of three consecutive measurements was obtained for each participant, with an acceptable intermeasurement variation defined as less than 0.5 m/s. When this criterion was not met, additional recordings were performed. The final PWV value corresponded to the mean of the three measurements with the smallest variability. Device-derived parameters included foot-to-e transit time (TT), foot-to-e PWV (m/s), and the standard deviations of TT and PWV. Arterial stiffness was evaluated using the pOpmètre device, a validated noninvasive reference method for PWV assessment [30].

### 2.6. Multicomponent Training Intervention

The intervention protocol was implemented in accordance with the framework previously described by Hernandez-Martinez, Valdés-Badilla, Cid-Calfucura, Vásquez-Carrasco, Marques and Magnani Branco [20] in a Brazilian population. The program consisted of three weekly MCT sessions and one weekly session dedicated to nutritional guidance and psychoeducational support.

All MCT sessions were conducted at university facilities and lasted approximately 60 min. The exercise program was designed to promote improvements in cardiorespiratory fitness, flexibility, muscle strength, mobility, motor coordination, and balance. MCT sessions were delivered three times per week and alternated between indoor and outdoor settings. Specifically, one weekly session was held indoors in a gym, while another was held outdoors in an open space. Exercise selection and progression were adapted to participants’ body mass characteristics to ensure appropriateness, safety, and individualized load management.

The MCT program commenced with a three-week neuromotor adaptation phase characterized by low training volume and intensity. Subsequently, exercise prescription followed a traditional linear periodization model [31,32], with a gradual and systematic increase in training volume and intensity across the remaining weeks, as detailed in Table 2. Each week, three MCT sessions involving exercises targeting large muscle groups, cardiorespiratory conditioning, and balance and flexibility training were conducted. Cardiorespiratory exercises were performed using a treadmill, stationary cycle ergometer, or rowing ergometer, selected according to individual preferences and functional capacity. Two weekly sessions emphasized resistance training combined with aerobic components, whereas the third session predominantly focused on neuromotor coordination and flexibility. This structured yet adaptable approach was designed to provide a comprehensive training stimulus while allowing individualization on the basis of participant needs.

Training load adjustments were guided by perceptual responses and self-selected effort. Exercise intensity was monitored during each session using the Rating of Perceived Exertion 0 to 10 (RPE session) scale, as proposed by Foster et al. [33], a method widely used in older populations to promote self-regulation, enhance safety, and support individualized load progression. In addition, the Rating of Perceived Recovery (RPR) scale developed by Laurent et al. [34] was administered prior to each session to assess recovery status relative to the preceding training bout. All participants received standardized instructions on the use of both perceptual scales before the intervention began.

Physiological safety monitoring included measurements of peripheral oxygen saturation (%SpO_2_) and blood pressure (systolic and diastolic) obtained immediately before and after each training session. Participants were instructed to report any symptoms such as dyspnea, excessive sweating, acute fatigue, or chest discomfort. SpO_2_ was continuously monitored during exercise, and the sessions were immediately discontinued if the %SpO_2_ values fell below 88%, indicating potential hypoxemia.

### 2.7. Nutritional Intervention

The nutritional education component aimed to enhance healthy eating behaviors among older women, drawing on the educational frameworks outlined by da Silva Oliveira and Silva-Amparo [35]. Two group-based nutritional education sessions, each lasting 30 min, were conducted prior to the MCT sessions.

The activities were carried out solely in a group format and focused on essential topics concerning physiological processes, quality of life, food literacy, specifically food labeling and processing, and methods for mitigating risk factors linked to noncommunicable diseases [36,37]. Participants did not receive personalized dietary prescriptions, nor were they closely monitored for caloric or protein intake. This approach was deliberately chosen to equip participants with practical knowledge and skills for autonomous health management, promoting sustainable behavioral change instead of temporary compliance with prescribed dietary regimens. The primary objective was to improve the understanding of macronutrients and micronutrients, hydration, and sodium consumption and to develop meal planning strategies that are appropriate for older people and are consistent with public health principles.

Attendance at sessions was documented to assess compliance. The educational curriculum included various topics, such as nutrition principles for chronic kidney disease; protein quality and leucine intake distribution; carbohydrate quality and glycemic control; dietary fat and cardiovascular health; sodium reduction and blood pressure management; hydration and fluid balance with diuretic considerations; calcium and vitamin D in bone health; vitamin B12, folate, iron, and anemia prevention; potassium and magnesium for cardiometabolic support; and dietary strategies for heart failure, with a focus on fluid and sodium management. The content covered meal planning, portion control through the plate method, food label interpretation focusing on added sugars, sodium, and trans fats, culinary skills that prioritize low-sodium and high-flavor techniques, cost-effective food purchasing, utilization of pantry and frozen staples, food safety and storage considerations for older people, oral health, dysphagia and texture modification, gastrointestinal health, including constipation and reflux, mindful eating practices, stress management and coping strategies, sleep quality and metabolic health, and the integration of physical activity with nutrition, particularly in relation to strength training and protein timing. Participant engagement and qualitative feedback were informally noted during the sessions; however, changes in dietary knowledge or behaviors were not formally evaluated as primary or secondary study outcomes.

### 2.8. Psychoeducation

The psychoeducation component consisted of one structured, group-based session per week, delivered for 30 min prior to the MCT sessions. This component was grounded in therapeutic and preventive approaches to mental health conditions, with the aim of providing knowledge and facilitating behavioral change in response to the psychological challenges of aging [38]. The intervention was organized as a progressive syllabus and delivered by trained psychologists.

The curriculum addressed a broad range of topics, beginning with group orientation elements (including session objectives, group norms, and therapeutic agreements), followed by foundational concepts related to mental health within a biopsychosocial framework. The core themes included stress physiology, stressors, and the window of tolerance; the interrelationship between thoughts, emotions, and behaviors; emotional awareness and labeling; the identification and restructuring of cognitive distortions; and the application of mindfulness and present-moment awareness in daily life. Additional content focused on emotion regulation strategies, distress tolerance, behavioral activation, routine planning, goal setting aligned with personal values, habit formation, motivation, adherence, and problem-solving skills.

Further sessions explored the relationships between nutrition, mood, emotional eating, anger, impulsivity, and sleep hygiene, as well as stress management techniques emphasizing safety, stability, grounding, and de-escalation. Topics related to grief and loss, chronic pain and mind–body integration, substance use and harm reduction, anxiety management through psychoeducation and gradual exposure, depressive symptoms and the cycle of inactivity, mood monitoring and early warning signs, and early identification of psychotic symptoms were also included. Trauma-informed principles, including anchoring, safety, and stabilization strategies, were integrated throughout the program.

Although formal psychometric outcomes were not evaluated as primary or secondary endpoints, participant engagement and qualitative feedback during group discussions were continuously observed by the facilitating psychologists, indicating the perceived relevance and acceptability of the psychoeducational intervention.

### 2.9. Statistical Analysis

Statistical analyses were conducted using GraphPad Prism software (version 9.0). Descriptive statistics are presented as the means and standard deviations. The Shapiro–Wilk test was used to evaluate the normality of the data. Two-factor mixed ANOVA with repeated measures was employed to evaluate the intervention effects over time and across various nutritional status groups. This model evaluated the interactions between time and group for BFP, FFM, visceral fat level, HDL-c, LDL-c, total cholesterol, triglycerides, glucose, and PWV.

Following the identification of a significant time × group interaction, a Bonferroni-adjusted post hoc analysis was performed to assess within-group differences (pre- vs. post-intervention) and between-group comparisons (normal weight vs. overweight vs. obese). The interaction effects were measured using partial eta squared (ηp^2^) [39], where values of 0.01, 0.06, and 0.14 corresponded to small, moderate, and large effects, respectively [39]. Effect sizes for pairwise comparisons were calculated using Cohen’s d, adhering to established thresholds of ≥0.2 (small), ≥0.5 (moderate), and ≥0.8 (large) [40]. All analyses were performed using a statistical significance threshold set at an alpha level of 0.05.

## 3. Results

Table 3 summarizes the pre- and postintervention outcomes for the variables assessed during the MCT program in older women classified as normal weight, overweight, or obese. A notable interaction between time and group regarding BFP and visceral fat level was observed using a two-factor mixed ANOVA (*p* < 0.001). No significant interaction effects were detected for FFM, lipid profile, fasting glucose, or arterial stiffness variables when the data were categorized by nutritional status.

The results of the effect sizes and magnitude of change in older women according to nutritional status (normal weight, overweight, and obese) for the variables analyzed for body composition, lipid profile, fasting glucose, and PWV are presented in Table 4.

At the individual level, favorable changes in body composition were observed following the intervention. Significant reductions in BFP were identified in overweight and obese older women (*p* < 0.01), with effect sizes ranging from moderate to large (ES = 0.55–1.27). The magnitude of BFP reduction was greater in the overweight group than in the normal-weight group (*p* < 0.01) and was similarly greater in the obese group than in the normal-weight group (*p* < 0.001).

Reductions in visceral fat level were observed across all nutritional status groups, with small to moderate effect sizes (ES = 0.38–0.44). Comparisons between groups indicated that the normal-weight group had greater reductions in visceral fat level than the overweight group did (*p* < 0.05), whereas compared with the normal-weight group, both the obese and overweight groups had significantly greater reductions (*p* < 0.001).

**Figure 2:**
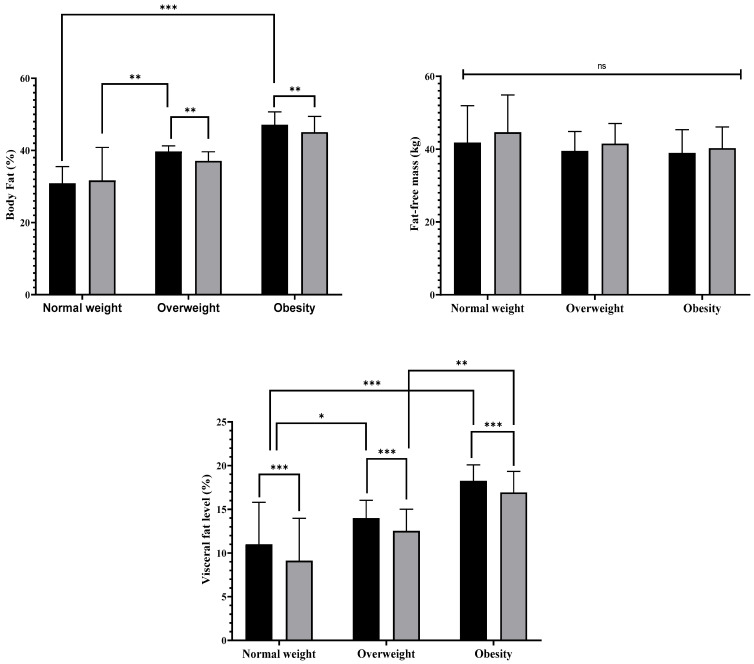
Changes in body composition according to nutritional status after 12 weeks of multicomponent training combined with multiprofessional intervention in older Brazilian women. Note: * = *p* < 0.05; ** = *p* < 0.01; *** = *p* < 0.001; ns: not significant.

In contrast, no statistically significant changes in FFM were detected in any group (*p ≥* 0.05), despite effect sizes ranging from trivial to small (ES = 0.01–0.27). These outcomes are illustrated in Figure 2.

**Figure 3:**
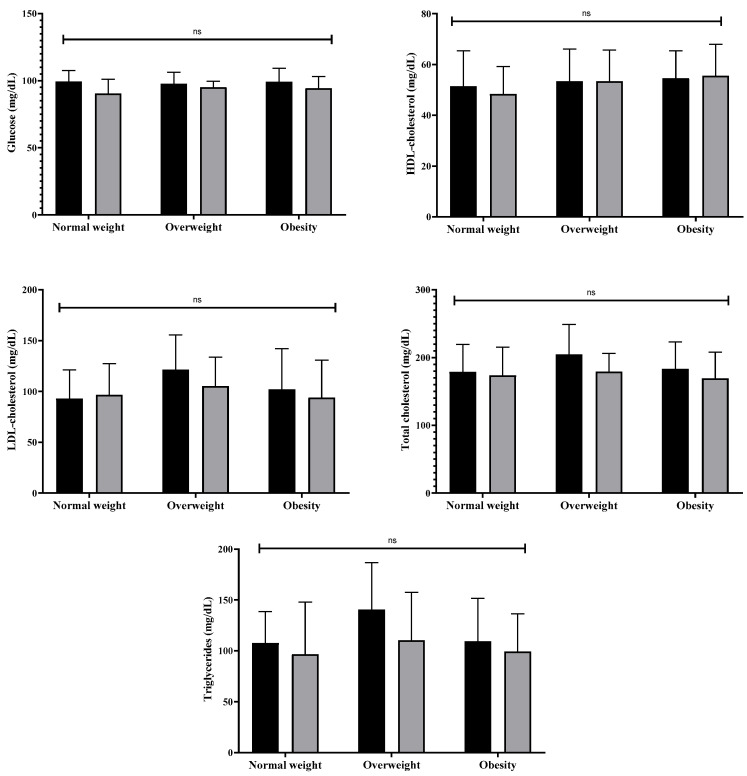
Changes in the lipid profile and fasting glucose according to nutritional status after 12 weeks of multicomponent training combined with multiprofessional intervention in older Brazilian women. Note: ns = not significant.

With respect to the lipid profile and fasting glucose levels (HDL-c, LDL-c, total cholesterol, triglycerides, and glucose levels), no statistically significant differences (*p* ≥ 0.05) were reported in any of the groups analyzed individually (ES = 0.08 to 0.64; 1.87% to 21.5%) or in the intergroup analysis. These results are presented in Figure 3.

**Figure 4:**
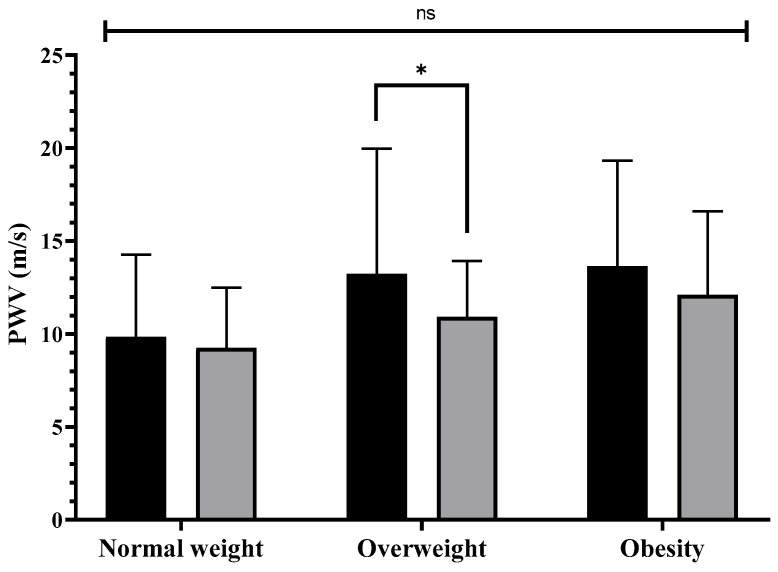
Changes in pulse wave velocity according to nutritional status after 12 weeks of multicomponent training combined with multiprofessional intervention in older Brazilian women. Note: PWV = pulse wave velocity; * = *p* < 0.05; ns: not significant.

In contrast, significant improvements (*p* < 0.05) were observed in individual PWV results in overweight older women (ES = 0.44; 17.4%). However, no significant differences were found between the groups (*p* ≥ 0.05). These results are presented in Figure 4.

## 4. Discussion

The present study examined the effects of an MCT program integrated with a multiprofessional intervention on body composition, lipid profiles, fasting glucose levels, and arterial stiffness in Brazilian older women stratified by nutritional status. These findings indicate that the combined intervention elicited meaningful improvements in selected body composition and vascular parameters. Specifically, significant reductions in BFP were observed among participants classified as overweight or obese, whereas decreases in visceral fat level were evident across all nutritional status groups. In addition, a significant improvement in arterial stiffness, as reflected by a reduction in PWV, was detected in overweight older women. Conversely, the intervention did not result in statistically significant changes in FFM or in lipid profiles or fasting glucose levels, including HDL-c, LDL-c, total cholesterol, triglycerides, or glucose levels, regardless of nutritional status.

### 4.1. Body Composition

Significant reductions in BFP were observed in older women classified as overweight or obese, whereas decreases in visceral fat level were evident across all nutritional status categories, including those with normal weight. These results align with those of previous studies conducted by Travieso, Ortiz, Abud, Villalba, Junqueira, Gomes, Marchini and Freitas [19], who reported notable reductions in BFP following a 12-week MCT program in Brazilian older people with obesity compared with an inactive control group. Pleticosic-Ramírez, Mecías-Calvo, Arufe-Giráldez and Navarro-Patón [21] reported notable decreases in total and visceral adiposity after six months of medium-chain triglyceride intervention in Chilean adults with obesity. The effectiveness of MCT in reducing adiposity is attributed to its holistic approach, which includes resistance, aerobic, balance, and agility training modalities.

The multimodal structure likely increased overall energy expenditure and enhanced fatty acid oxidation in intramuscular and visceral depots, which are particularly important targets in older people [41]. The moderate-intensity resistance training component likely contributed to the preservation of FFM, thereby facilitating a sustained negative energy balance during the intervention period [42]. The concurrent incorporation of nutritional education and psychoeducational strategies likely improved dietary quality, self-regulation, and awareness of the importance of physical activity in promoting healthy aging and quality of life.

The absence of notable changes in BFP among normal-weight participants can be attributed to the limited sample size in this subgroup (n = 8), which likely reduced the statistical power to detect modest intervention effects over 12 weeks. Moreover, differences in metabolic flexibility and the lipolytic responsiveness of adipose tissue among individuals may result in diminished fat loss responses in normal-weight older women, as suggested by previous studies on metabolism and aging [43,44].

No statistically significant increases in FFM were observed across any nutritional status group. Similar findings concerning older women have been documented in prior studies. Hernandez-Martinez et al. [45] and Valdés-Badilla et al. [46] reported no significant improvements in FFM among older women in two independent studies: the first compared an eight-week MCT program with elastic band training (*p* = 0.62), and the second compared MCT with a group based on Taekwondo (*p* = 0.17). Villareal, Smith, Sinacore, Shah and Mittendorfer [42] reported notable increases in FFM following three months of MCT, which included 36 training sessions for older people with obesity. These findings imply a potential dose–response relationship, indicating that an extended intervention duration and greater cumulative training volume may be necessary to achieve substantial hypertrophic adaptations. In this study, modest positive trends were observed; however, the magnitude of change was small (ES = 0.01–0.27; Δ = 0.24–6.69%).

The absence of statistical significance may be due to the short intervention period, which likely did not provide a sustained anabolic stimulus. Furthermore, while a nutritional education component was included, it lacked individualized dietary prescriptions and rigorous monitoring of energy and protein intake, both of which are essential factors influencing muscle protein synthesis and lean mass accretion [47]. Age-related anabolic resistance may diminish skeletal muscle responsiveness to moderate-intensity exercise, which requires increased mechanical loading and/or specific protein supplementation to achieve significant improvements in FFM [43,44]. Future interventions designed to enhance muscle adaptations in older women should extend beyond a 12-week duration and incorporate personalized nutritional strategies. The combination of structured exercise and sufficient protein intake is essential for enhancing muscle remodeling and maintaining lean mass in aging individuals [48].

### 4.2. Lipid Profile and Fasting Glucose

In the present study, no statistically significant changes were detected in HDL-c, LDL-c, total cholesterol, triglyceride, or glucose concentrations following 12 weeks of MCT combined with nutritional and psychoeducational counseling. Although small changes were observed across most metabolic markers (ES = 0.08–0.64), these should be interpreted with caution, as the absence of statistical significance prevents drawing definitive conclusions regarding the effectiveness of the intervention. Furthermore, between-group comparisons indicated moderate-to-large effect sizes when normal-weight and overweight participants were compared, particularly for total cholesterol (*d* = 0.63; +14.4%), LDL-c (*d* = 0.90; −30.6%), and triglycerides (*d* = 0.83; −30.5%). However, these effect sizes should be considered exploratory and descriptive, as they are not supported by statistically significant differences. These findings suggest a more pronounced metabolic responsiveness among overweight older women. These findings differ from those reported by Subías-Perié, Navarrete-Villanueva, Fernández-García, Moradell, Lozano-Berges, Gesteiro, Pérez-Gómez, Ara, Gómez-Cabello, Vicente-Rodríguez and Casajús [18], who reported significant increases in HDL-c after six months of MCT; by Hejazi et al. [49], whose meta-analysis revealed significant reductions (*p* < 0.05) in LDL-c and total cholesterol in favor of concurrent training compared with aerobic, resistance, and tai chi training, with effects being more consistent in interventions lasting ≥12 weeks, and by Gargallo et al. [50], who reported significant improvements (*p* < 0.006) in blood glucose, triglycerides, and total cholesterol after 20 weeks of MCT in older women with metabolic syndrome.

From a physiological perspective, the combination of strength and aerobic stimuli in MCTs may promote favorable changes in lipid metabolism when sustained over longer periods (>12 weeks), increasing the lipoprotein lipase (LPL) activity responsible for LDL-c particle clearance and improving fatty acid uptake and oxidation [51,52]. Additionally, the anti-inflammatory effects of regular physical activity may reduce LDL-c oxidation and improve HDL-c functionality, contributing to a more cardioprotective lipid profile [53,54]. However, the relatively short duration of our intervention (12 weeks) likely did not provide a sustained lipid turnover stimulus sufficient to significantly activate reverse cholesterol transport, a process that typically requires greater cumulative training volume and higher weekly energy expenditure [54,55]. These findings are supported by previous studies [55,56] showing that exercise volume and intensity can strongly influence HDL-c levels. In particular, weekly energy expenditures between 1200 and 2200 kcal have been proposed as an optimal dose to induce meaningful increases in HDL-c [55]. Moreover, several studies have suggested that compared with moderate exercise, vigorous-intensity exercise may be more effective for improving HDL-c levels [57,58]. In this context, the MCT protocol used in our study was adapted to individual functional capacities in older women and was not performed at vigorous intensity. Combined with the lack of control over participants’ weekly energy expenditure, this may explain the less pronounced effects on HDL-c levels. Finally, although our MCT included a nutritional intervention that likely encouraged healthier eating habits, the absence of individualized dietary planning and monitoring of unsaturated fat and omega-3 fatty acid intake may have limited the magnitude of the lipid response [59].

With respect to blood glucose, previous studies have reported that the effect of exercise on older people depends on the duration of the intervention, the amount of active muscle mass involved, and the magnitude of cumulative energy expenditure [18,50]. In the present study, although MCT predominantly combines strength and aerobic stimuli, its moderate intensity and relatively short duration (12 weeks) may have been insufficient to induce substantial adaptations in GLUT-4 translocation or muscle hexokinase activity, both of which are key processes for improving insulin sensitivity [50,60]. From a physiological standpoint, older people also exhibit age-related anabolic resistance, characterized by blunted activation of the AMPK, PI3K, and Akt signaling pathways in response to exercise, which may attenuate the expected improvements in glucose regulation despite consistent training adherence [43,61]. Accordingly, the absence of statistically significant changes in glucose should not be interpreted as evidence of metabolic improvement, but rather as a lack of detectable effect under the conditions of the present study. Nevertheless, the maintenance of stable glucose and lipid concentrations during the intervention period may be interpreted as a protective effect against the progressive metabolic decline typically observed with sedentary aging [51].

### 4.3. Arterial Stiffness

Significant improvements (meaning reductions) in PWV were observed in overweight older women, whereas no significant changes were detected in those with normal weight or obesity. These findings suggest a more favorable vascular response in older women with moderate excess weight, possibly attributable to a greater initial margin for improvement or enhanced hemodynamic sensitivity to exercise stimuli [62]. Among women with obesity, although the changes did not reach statistical significance, a trend toward reduced PWV was observed, with a moderate effect size (*d* = 0.74) and an average reduction of 38.3% compared with older women of normal weight. However, given the lack of statistical significance, these changes should be interpreted cautiously and may reflect variability or insufficient statistical power rather than true physiological adaptations. Therefore, these findings should be considered preliminary and hypothesis-generating rather than conclusive evidence of intervention effectiveness [63,64]. In contrast, the meta-analysis by Ashor, Lara, Celis-Morales and Mathers [22], which included young, middle-aged, and older people with obesity, reported significant decreases (*p* < 0.001) in arterial stiffness measured by PWV in favor of aerobic exercise compared with strength or concurrent training. Similarly, Li, Lv, Su, You and Yu [63] reported significant reductions (*p* = 0.002) in PWV among middle-aged and older people following aerobic exercise programs, with effects being more pronounced in interventions of higher intensity (vigorous vs. moderate).

From a physiological perspective, the improvements in PWV observed among overweight older women may be explained by greater nitric oxide (NO) bioavailability induced by exercise, which enhances endothelium-dependent vasodilation and reduces arterial wall tension [63,65]. The aerobic component of MCT likely promotes more stable laminar blood flow and repeated endothelial shear stress, stimulating the expression of endothelial nitric oxide synthase (eNOS) and promoting a more elastic vascular tone [63,65]. In this context, a study analyzing the effects of high-intensity interval training on PWV in older people reported that each 10 mL·min^−1^·kg^−1^ increase in maximum oxygen consumption (VO_2_max) was associated with a 0.8 m/s decrease in PWV, a meaningful change considering that a 1 m/s increase in central PWV has been linked to a 15% higher risk of all-cause mortality in older people [65]. In parallel, the moderate-intensity resistance component in our study may have contributed to lower systolic blood pressure and reduced chronic hemodynamic stress, thereby diminishing mechanical loading on arterial walls [62]. Additionally, MCTs may partially attenuate low-grade systemic inflammation associated with aging and excess adiposity by reducing proinflammatory cytokine production (IL-6, TNF-α) and vascular oxidative stress mechanisms, which can improve arterial distensibility and contribute to reduced stiffness [65]. However, these processes were not directly measured in our study; therefore, this interpretation should be considered hypothetical and based on previous evidence.

### 4.4. Limitations and Strengths

This study has several limitations that must be acknowledged in the interpretation of its findings. The lack of personalized dietary prescriptions and insufficient monitoring of total energy intake may have diminished the extent of metabolic and body composition adaptations observed. The 12-week intervention period may have been inadequate to induce structural changes in FFM or to achieve lasting improvements in lipid profile parameters. The lack of stringent regulation of external factors, such as medication use, regular physical activity, and daily dietary habits, may have contributed to the variability in physiological responses to the MCT intervention.

Despite these limitations, various strengths enhance the significance of the current study. The intervention utilized a controlled, multiprofessional design that combined exercise training, nutritional education, and psychoeducational support, demonstrating a comprehensive approach to health promotion among older people. The inclusion of participants from various nutritional status categories (normal weight, overweight, and obesity) facilitated the analysis of differential responses to MCT. The use of validated and reliable methods for assessing body composition, lipid profiles, fasting glucose levels, and PWV enhances the methodological rigor and external validity of the findings. The integration of MCTs and multiprofessional support is consistent with current guidelines aimed at enhancing cardiometabolic and functional health in older people.

### 4.5. Practical Applications

Promotion of cardiometabolic health: MCTs performed three times per week, when combined with nutritional education and psychoeducational support, constitute a feasible and safe strategy for improving cardiovascular and metabolic health in older women across different nutritional status categories.

Reduced adiposity: MCT is effective at reducing BFP and visceral fat level, particularly among overweight and obese older women, supporting its use as a nonpharmacological intervention for the management of excess adiposity in aging populations.

Improvement in arterial stiffness: The observed reductions in PWV among overweight participants suggest that MCTs can increase vascular compliance and endothelial function, even within relatively short intervention periods.

Implications for program design: To maximize adaptations in lipid profiles and FFM, future interventions should consider extending the program duration beyond 12 weeks, increasing cumulative weekly energy expenditure, and incorporating individualized nutritional strategies when feasible.

Translation to clinical practice: The integration of MCTs within multiprofessional health promotion programs offers a cost-effective, scalable, and time-efficient model for preserving cardiometabolic health and functional capacity throughout the aging process.

## 5. Conclusions

A 12-week MCT program integrated with multiprofessional support was associated with significant reductions in BFP among overweight and obese older women, whereas decreases in visceral fat level were observed across normal-weight, overweight, and obese groups. In addition, significant improvements in PWV, indicating reduced arterial stiffness, were identified in overweight participants. Collectively, these findings underscore the clinical relevance of MCTs combined with multiprofessional intervention as a nonpharmacological strategy with considerable potential to mitigate cardiometabolic risk associated with excess adiposity in Brazilian older women.

## Figures and Tables

**Table 1:** Basic characteristics of the sample.

	Normal Weight (n = 8)	Overweight (n = 13)	Obesity (n = 15)
Age (years)	69.2 ± 7.21	72.1 ± 5.34	70.3 ± 4.56
Body weight (kg)	65.6 ± 9.46	65.4 ± 4.6	74.2 ± 4.9
Height (m)	1.57 ± 4.2	1.48 ± 3.1	1.58 ± 2.4
Body mass index (kg/m^2^)	26.0 ± 2.62	29.9 ± 3.1	29.7 ± 5.03
Body fat percentage (%)	30.9 ± 4.61	39.7 ± 1.54	47.1 ± 3.59
Fat free mass (kg)	41.8 ± 5.1	39.4 ± 5.24	40.2 ± 5.77

Values are mean ± sd.

**Table 2:** Periodization scheme of the multicomponent training intervention.

Program	Physical Exercise	Training Session	Frequency (weekly)	Session Time	Set	Time	Rest	Cadence
MCT	Inside	A (9 exercises)	1	≈60 min	2	40 s	20 s	4-0-2-0
		B (8 exercises)	1	≈60 min	3	30 s	30 s	Slowly 1-0-1-0
	Outside	A (6 exercises)	1	≈60 min	2	40 s	20 s	4-0-2-0
		B (7 exercises + 10 min walking)	1	≈60 min	3	30 s	45 s	1-concentric-1-eccentric

Note: MCT = multicomponent training.

**Table 3:** Time x group interaction in the analyzed variables of multicomponent training combined with multiprofessional intervention on body composition, lipid profile, fasting glucose, and arterial stiffness in older women according to nutritional status.

	Normal Weight (n = 8)		Overweight (n = 13)		Obesity (n = 15)		Group x Time		ηp^2^
	Baseline	12 Weeks	Baseline	12 Weeks	Baseline	12 Weeks	F Value	*p* Value	
BFP (%)	47.1 ± 3.19	45.0 ± 4.34	39.7 ± 1.59	37.0 ± 2.53	30.9 ± 4.61	31.7 ± 9.14	39.4	0.000	0.70
FFM (kg)	41.8 ± 10.1	44.6 ± 10.2	39.4 ± 5.24	41.5 ± 5.50	40.2 ± 5.77	40.3 ± 5.66	0.53	0.59	0.03
Visceral fat level (L)	11.0 ± 4.81	9.12 ± 4.85	14.0 ± 2.04	12.5 ± 2.47	18.2 ± 2.83	16.1 ± 4.19	19.5	0.000	0.54
Fasting glucose (mg/dL)	99.5 ± 8.0	90.5 ± 2.6	97.7 ± 8.41	95.0 ± 4.40	99.1 ± 10.0	94.2 ± 8.76	0.15	0.86	0.00
HDL-c (mg/dL)	51.4 ± 13.1	48.3 ± 10.8	53.4 ± 12.6	54.4 ± 12.1	54.5 ± 10.8	55.6 ± 12.3	0.57	0.56	0.03
LDL-c (mg/dL)	92.9 ± 28.3	96.7 ± 30.5	121.4 ± 34.2	105.2 ± 28.6	102.1 ± 40.0	93.8 ± 36.9	1.07	0.35	0.61
Total cholesterol (mg/dL)	178.8 ± 40.6	173.8 ± 41.5	204.6 ± 40.3	179.3 ± 26.9	183.3 ± 39.7	169.4 ± 38.2	0.74	0.48	0.04
Triglycerides (mg/dL)	107.7 ± 30.8	96.6 ± 51.2	140.6 ± 46.1	110.3 ± 47.2	109.3 ± 42.3	99.4 ± 36.8	0.45	0.64	0.01
PWV (m/s)	9.83 ± 4.42	9.25 ± 3.24	13.2 ± 6.74	10.9 ± 2.99	13.6 ± 5.67	12.1 ± 4.48	1.60	0.21	0.08

Note: data are expressed as mean ± standard deviation; BFP = body fat percentage; FFM = fat-free mass; HDL-c = high-density lipoprotein cholesterol; LDL-c = low-density lipoprotein cholesterol; PWV = pulse wave velocity; ηp^2^ = eta squared; mg/dL: milligrams per deciliter; L: liters; kg: kilograms; %: percentage; m/s: meters per second.

**Table 4:** Effect size and magnitude of change in the analyzed variables of multicomponent training combined with multiprofessional intervention on body composition, lipid profile, fasting glucose, and arterial stiffness in older women according to nutritional status.

	BFP	FFM (kg)	Visceral Fat Level (L)	Glucose (mg/dL)	HDL-c (mg/dL)	LDL-c (mg/dL)	Total Cholesterol (mg/dL)	Triglycerides (mg/dL)	PWV (m/s)
Normal Weight vs. Overweight	*d* = 2.93 ^d^ 15.7%	*d* = 0.29 ^b^ 5.02%	*d* = 0.84 ^d^ 27.27%	*d* = 0.21 ^b^ 1.80%	*d* = 0.15 ^a^ 3.89%	*d* = 0.90 ^d^ 30.67%	*d* = 0.63 ^c^ 14.42%	*d* = 0.83 ^d^ 30.54%	*d* = 0.59 ^c^ 34.28%
Normal Weight vs. Obesity	*d* = 4.08 ^d^ 34.39%	*d* = 0.19 ^a^ 3.82%	*d* = 1.82 ^d^ 65.45%	*d* = 0.04 ^a^ 0.40	*d* = 0.25 ^b^ 6.03%	*d* = 0.26 ^b^ 9.90%	*d* = 0.11 ^a^ 2.51%	*d* = 0.04 ^a^ 1.48%	*d* = 0.74 ^c^ 38.35%
Overweight vs. Obesity	*d* = 2.55 ^d^ 22.16%	*d* = 0.14 ^a^ 2.03%	*d* = 1.70 ^d^ 30%	*d* = 0.15 ^a^ 1.43%	*d* = 0.09 ^a^ 2.05%	*d* = 0.51 ^c^ 15.89%	*d* = 0.53 ^c^ 10.41%	*d* = 0.70 ^c^ 22.26%	*d* = 0.06 ^a^ 3.03%

Note: BFP = body fat percentage; FFM = fat-free mass; HDL-c = high-density lipoprotein cholesterol; LDL-c = low-density lipoprotein cholesterol; PWV = pulse wave velocity; mg/dL: milligrams per deciliter; L: liters; kg: kilograms; %: percentage; m/s: meters per second; vs: versus; *d* = effect size; a = nonsignificant effect (<0.20); b = small effect (0.20–0.49); c = moderate effect (0.50–0.79); d = large effect (≥ 0.80).

## Data Availability

The data presented in this study are available upon request from the corresponding author (specify the reason for the restriction).
